# Supplementary material for: A Likelihood Approach for Real-Time Calibration of Stochastic Compartmental Epidemic Models
Source: PLoS Comput Biol. 2017 Jan 17;13(1):e1005257. doi: 10.1371/journal.pcbi.1005257 (PMC5240920; doi:10.1371/journal.pcbi.1005257)
Supplement: S1 File — (TAR.GZ) [file pcbi.1005257.s014.tar.gz › HSPH_Online-SI-Revision/IPoi09/n10-mild/n10-mild_BENCH_summary.pdf]

peak -8	R0	$1/\theta_2$	inf	inflog	newly	newlylog	Reff	AttackRate
mean	28.6	66.8	276.1	0.5	348.1	0.6	34.1	58.8
median	16.5	47.2	250.2	0.5	274.6	0.6	20.6	49.
Quantile .1	3.7	22.1	83.	0.3	85.5	0.4	11.2	15.2
Quantile .9	76.4	169.1	476.3	0.8	648.	0.8	82.1	118.5
10	15	15						
length	50							

peak -4	R0	$1/\theta_2$	inf	inflog	newly	newlylog	Reff	AttackRate
mean	25.5	63.3	192.5	0.4	123.4	0.4	26.3	54.
median	17.9	33.5	168.	0.4	119.2	0.3	20.8	51.1
Quantile .1	4.5	7.7	53.	0.2	62.1	0.2	12.5	13.4
Quantile .9	50.5	173.	307.8	0.6	180.9	0.5	43.4	85.3
0	6	3						
length	50							

peak	R0	$1/\theta_2$	inf	inflog	newly	newlylog	Reff	AttackRate
mean	52.6	124.4	318.7	0.6	97.	0.3	32.3	90.3
median	45.9	86.1	268.2	0.6	88.1	0.3	28.9	86.1
Quantile .1	27.1	16.3	122.4	0.3	50.3	0.2	21.	50.9
Quantile .9	89.1	286.1	505.	0.8	150.1	0.4	53.2	124.8
0	0	0						
length	50							

peak +4	R0	$1/\theta_2$	inf	inflog	newly	newlylog	Reff	AttackRate
mean	50.8	112.9	350.5	0.6	113.6	0.3	35.2	91.
median	49.7	101.1	310.2	0.6	96.7	0.3	35.1	90.8
Quantile .1	32.9	34.5	121.6	0.3	61.8	0.2	27.7	63.5
Quantile .9	71.9	202.2	660.9	0.9	183.3	0.4	41.8	118.1
0	1	0						
length	50							

peak +8	R0	$1/\theta_2$	inf	inflog	newly	newlylog	Reff	AttackRate
mean	51.	112.8	31713.8	0.6	524.	0.4	41.	92.
median	52.6	114.	290.3	0.5	107.	0.3	42.2	88.2
Quantile .1	32.9	37.6	78.7	0.3	55.3	0.2	29.4	64.4
Quantile .9	64.4	182.9	1574.9	1.1	635.8	0.7	48.8	120.7
0	0	0						
length	50							
